# An activity‐based bioprobe differentiates a novel small molecule inhibitor from a LOXL2 antibody and provides renewed promise for anti‐fibrotic therapeutic strategies

**DOI:** 10.1002/ctm2.572

**Published:** 2021-11-06

**Authors:** Alison Findlay, Craig Turner, Heidi Schilter, Mandar Deodhar, Wenbin Zhou, Lara Perryman, Jonathan Foot, Amna Zahoor, Yimin Yao, Ross Hamilton, Mary Brock, Christina Raso, Jessica Stolp, Marie Galati, Dieter Hamprecht, Brett Charlton, Wolfgang Jarolimek

**Affiliations:** ^1^ Pharmaxis Frenchs Forest New South Wales Australia; ^2^ Quanterix Billerica Massachusetts USA

Dear Editor,

In this study, measurement of target inhibition of lysyl oxidase‐like 2 (LOXL2) in a high throughput manner from tissue lysates and blood was achieved by the tailored design of an activity‐based probe (ABP), PXS‐5878. Effective inhibition of LOXL2 enzymatic activity in vitro, in vivo and in a phase 1 study in healthy humans was demonstrated by a novel small molecule, PXS‐5338. In contrast to the encouraging results obtained with PXS‐5338, our study also revealed the inability of a LOXL2 antibody (AB0023) to effectively inhibit the target, thereby providing a plausible explanation for the failure of the corresponding humanised antibody, simtuzumab, in the clinic.

Lysyl oxidases are a family of five enzymes critically responsible for the formation of cross‐linked collagen and elastin, the hallmarks of fibrosis and stroma.^1^, ^2^ One member in particular, LOXL2, has excellent pre‐clinical target validation, is upregulated in various fibrotic diseases and cancer,^3^ and acts as a biomarker for disease severity and progression in humans.^4^, ^5^ Despite overwhelming target rationale, the failure of the LOXL2 antibody simtuzumab to achieve positive clinical endpoints^6^, ^7^ has undoubtedly hampered progress in the field and cast doubt over the validity of LOXL2 inhibition as a viable therapeutic approach. This may now be overcome with the use of an ABP to interrogate the extent of target engagement achieved by the small molecule LOXL2 inhibitor PXS‐5338, providing crucial information lacking from previous trials. The ability to confirm sufficient target engagement in humans is of vital importance for successful clinical candidate progression, potentially reducing attrition rates due to inappropriate dosing regimens.

PXS‐5878 (Figure 1A), a novel biotinylated ABP, was designed to potently and irreversibly bind to unoccupied lysyl oxidase active sites (Figure 1B,C). Binding is blocked by pre‐treatment with a lysyl oxidase inhibitor (Figure 1D,E) and the irreversible nature of inhibition is lost when PXS‐5878 is modified (Figure 1C), confirming its mechanism‐based mode of action. Importantly, the ABP can be combined with Simoa® bead technology^8^ to determine enzymatic activity in low volumes. Specifically, an LOXL2 capturing antibody is used in combination with either PXS‐5878 to measure activity or a second anti‐LOXL2 antibody to measure protein concentration (Figure S2).

**FIGURE 1:**
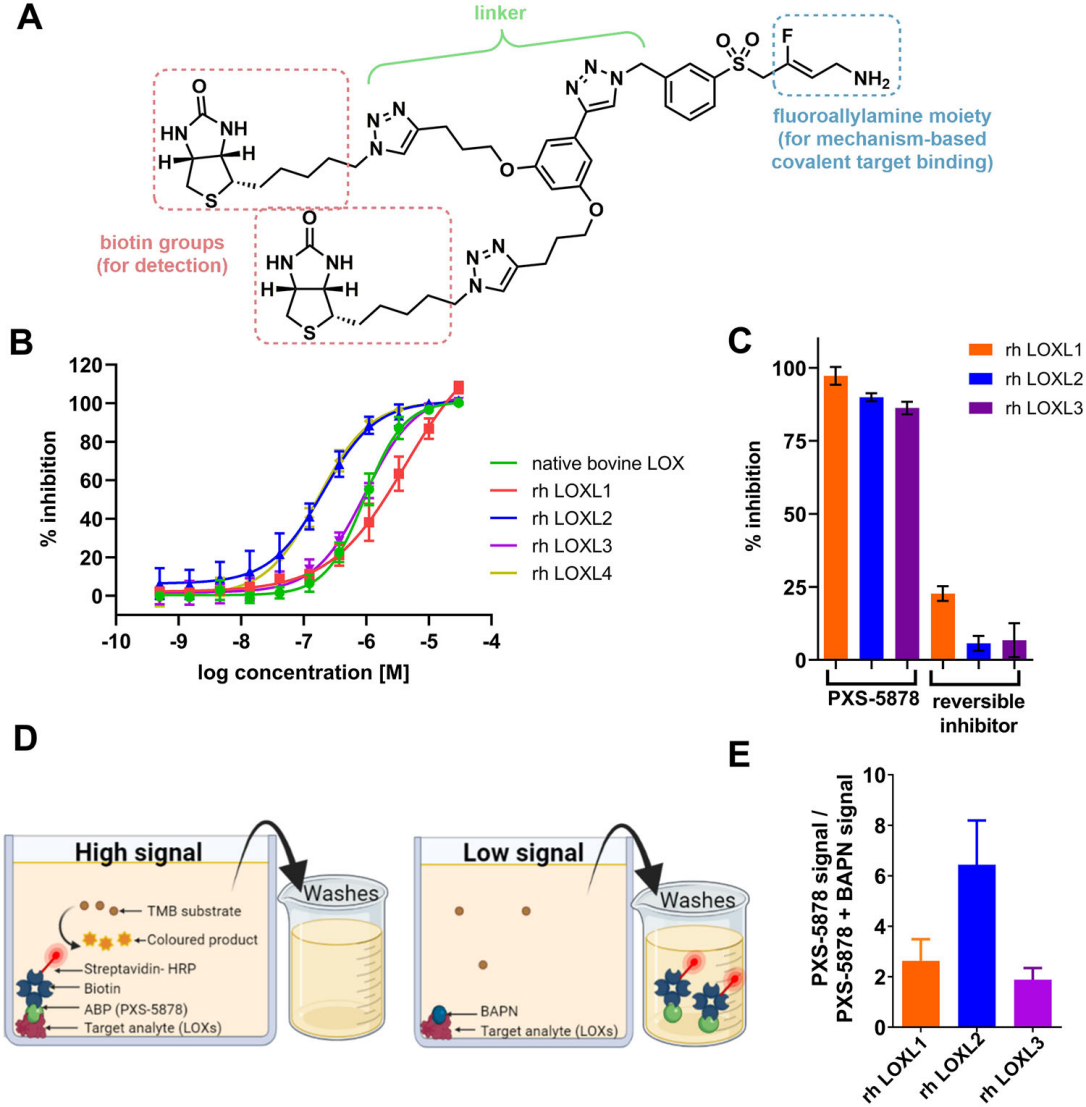
Characteristics of the activity‐based probe (ABP) PXS‐5878. (A) Chemical structure of PXS‐5878, with functional subunits denoted with different colours. The fluoroallylamine moiety (blue) was designed to interact with the lysine tyrosylquinone (LTQ) co‐factor within the lysyl oxidase active sites. An extended linker (green) was incorporated to provide optimal separation between the reactive functional group and the tag, avoiding unfavourable steric hindrance. Biotin was selected as a tag, with higher sensitivity found to be achieved using a double tag (red), resulting in PXS‐5878 as the preferred ABP. (B) Concentration‐dependent inhibition of recombinant human (rh) lysyl oxidase‐like 1‐4 (LOXL1‐4) and native bovine LOX in a standard Amplex Red assay with 30 min pre‐incubation; *n* = 3–4 independent experiments, standard error of mean (SEM). Native bovine LOX was used as a surrogate for human LOX as both enzymes have similar pharmacology for fluoroallylamines.^2^, ^9^ (C) Irreversible nature of PXS‐5878 enzyme inhibition confirmed using a jump dilution assay. Enzyme (rh LOXL1, rh LOXL2 or LOXL3) was pre‐incubated with 10 × IC_50_ of PXS‐5878 for 30 min, then diluted 100x and remaining activity assessed by Amplex Red assay. Little to no recovery in enzyme activity was seen for PXS‐5878. In contrast, enzyme activity almost fully recovered upon inhibition by an analogue of comparable potency but lacking the fluoro group (reversible inhibitor, Figure S1), confirming the importance of the putative leaving group for the irreversible binding profile. Each value is an average of three experiments. (D) Schematic representation of the assay development to detect lysyl oxidase activity by PXS‐5878 using a direct enzyme‐linked assay with lysyl oxidases as the target analyte. HRP: horseradish peroxidase; TMB: 3,3′,5,5′‐tetramethylbenzidine, a chromogenic substrate. After target analyte is bound to the plate, PXS‐5878 was incubated for 60 min in the presence or absence of a pan‐LOX inhibitor (β‐aminopropionitrile, [BAPN]) and streptavidin‐HRP is then added. After incubation the plate is washed and TMB added to measure the amount of bound ABP/streptavidin/HRP. This leads to a high signal. Application of BAPN to block the enzymatic site prevents PXS‐5878 from binding, and it is consequently washed off the plate, resulting in a low signal. In the final Simoa® assay, the enzyme is bound by the antibody to the bead. (E) Detection of enzymatically active lysyl oxidases by PXS‐5878. rh LOXL1, LOXL2 or LOXL3 were immobilised on the surface of a microtiter plate and processed as described in (D). Optical density in the absence of the pan‐LOX inhibitor BAPN (high signal) was divided by the value in the presence of BAPN (low signal). Low signal was 1.81 ± 0.77 (*n* = 8) times larger than blank controls. Two independent replicates were performed (total *n* = 4–5 for each enzyme).

PXS‐5338 (Figure 2A), related to the anti‐fibrotic LOXL2 inhibitor PXS‐5153,^9^ encompasses several important drug‐like features inevitably lacking from an antibody such as AB0023, including excellent tissue penetration (Figure 2B) and good oral bioavailability. It is a potent, selective, fast acting mechanism‐based inhibitor (Figure S3) of recombinant human (rh) LOXL2, with an IC_50_ of 35 nM after 30 min pre‐incubation and full inhibition achieved at concentrations above 300 nM (Figure 2C,D) in a standard assay using putrescine or collagen (Figure S4) as substrate. In contrast (but in line with published literature^3^) AB0023 showed only partial inhibition of rh LOXL2 activity, with approximately 50% reduction at 1 μM (Figure 2E; Figure S4). Small molecule, selective LOXL2 (PXS‐5338, Figure S3) or pan‐LOX inhibitors compete with substrate but ultimately occupy the enzymatic pocket, irrespective of whether the substrate is small (e.g., putrescine) or large (e.g., collagen). While AB0023 showed some inhibition of collagen oxidation, the isotype control antibody GS834298 displayed similar inhibition, indicative of an unspecific effect (Figure S4). Further studies with AB0023 were therefore limited to 300 nM.

**FIGURE 2:**
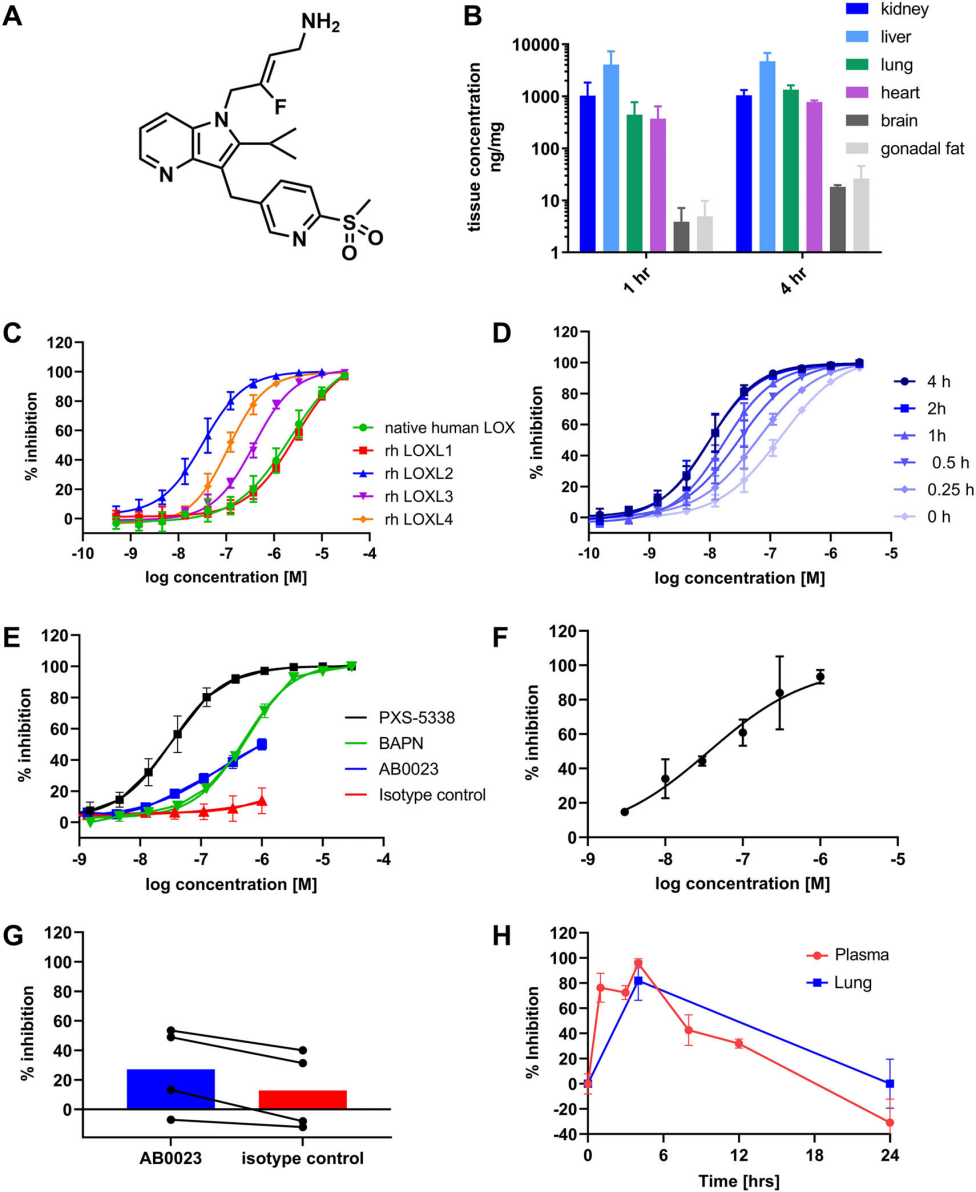
Characteristics of PXS‐5338 and inhibition of lysyl oxidase‐like 2 (LOXL2) enzymatic activity in vitro and in vivo. (A) Chemical structure of PXS‐5338. PXS‐5338 (like PXS‐5878) contains a fluoroallylamine to interact with the lysine tyrosylquinone (LTQ) co‐factor within the active site of LOXL2 and irreversibly inhibits the enzyme. The corresponding structure lacking the fluorine (des‐fluoro‐PXS‐5338, Figure S1) is a reversible inhibitor. (B) Tissue levels of PXS‐5338 measured 1 and 4 h after a single oral dose of 30 mg/kg in the rat. PXS‐5338 shows good penetration, with preferential distribution to the liver followed by the kidney, lung and heart. Average data from three different animals. (C) Concentration‐dependent inhibition of native human LOX and rh LOXL1‐4 in a standard Amplex Red assay with 30 min pre‐incubation; *n* ≥3 independent experiments, standard error of mean (SEM). (D) PXS‐5338 inhibits rh LOXL2 in a time‐dependent manner, with increasing potency observed upon longer incubation with the enzyme. LOXL2 activity data devised from Amplex Red assay using putrescine as substrate and varying pre‐incubation times; *n* ≥3 independent experiments, standard error of mean (SEM). 2 h and 4 h time points are superimposed suggesting that inhibition is complete after 2 h. PXS‐5338 is therefore considered a potent (IC_50_ 35 nM at rh LOXL2 with 30 min pre‐incubation) and fast acting LOXL2 inhibitor unlike PAT‐1251 (also known as GB2064).^10^ (E) Anti‐LOXL2 antibody AB0023 is a low potency, partial inhibitor of rh LOXL2 activity when tested in the standard Amplex Red assay using putrescine as a substrate as previously described.^3^ AB0023 and the corresponding isotype control antibody GS834298 were kindly provided by Gilead. Concentration‐response curves for the pan‐LOX inhibitor BAPN and LOXL2‐selective inhibitor PXS‐5338 are included for comparison. Data represented as mean ± SD with *n* = 5/concentration. (F) LOXL2 activity in human plasma is fully inhibited by a small molecule inhibitor PXS‐5338. To enable translation of preclinical data into the clinic, human plasma was incubated with different concentrations of PXS‐5338 for 30 min and the PXS‐5878/Simoa® platform used to measure the residual activity. PXS‐5338 inhibition of native LOXL2 protein in human plasma shows good agreement (IC_50 _=_ _37 nM) with the concentration response curve for rh LOXL2. Results are from five independent experiments using different human plasma sources (untreated samples from clinical trials). (G) LOXL2 activity in human plasma is not specifically inhibited by anti‐LOXL2 antibody AB0023. Using the same experimental set up as described for (F), human plasma was incubated with 300 nM of AB0023 or isotype control antibody GS834298 and the PXS‐5878/Simoa platform used to measure the residual activity of native LOXL2, with almost identical activity obtained for AB0023 and GS834298. Results are displayed as % inhibition, with control being the activity in the absence of an antibody. Results are from five independent experiments using different human plasma sources (untreated samples from clinical trials). (H) Inhibition of LOXL2 activity after administration of the LOXL2 inhibitor PXS‐5338 (30 mg/kg at *t* = 0) at different time point in rats. Data represented as mean ± SEM from at least two animals per time point.

In clinical studies with simtuzumab, the humanised version of AB0023, target engagement in human blood was not measured, triggering uncertainty about the lack of efficacy in humans. We therefore used ABP PXS‐5878 to confirm a robust inhibition of native human plasma LOXL2 by PXS‐5338, with an IC_50_ almost identical to that measured for rh LOXL2 (Figure 2F). In contrast, under identical conditions, AB0023 showed only minimal specific inhibition (Figure 2G).

The ability of PXS‐5338 to effectively inhibit LOXL2 in tissues, as well as the subsequent recovery of enzymatic activity following inhibition, was next assessed. Rats were orally dosed with PXS‐5338 (30 mg/kg) and the PXS‐5878/Simoa® platform used to measure enzymatic activity ex vivo. As in plasma, almost complete inhibition of LOXL2 activity in the lung occurred at 4 h (Figure 2H), confirming the tissue penetrating properties of PXS‐5338 as well as the suitability of plasma LOXL2 as a surrogate for tissue activity. Remarkably, no inhibition remained at 24 h, revealing the unexpectedly rapid turnover cycle of LOXL2 protein.

To confirm the therapeutic potential of the anti‐fibrotic PXS‐5338 (Figure S5) in humans, LOXL2 inhibition after oral administration was examined in a phase 1 study. LOXL2 protein concentration did not change significantly over time in the presence of PXS‐5338 when compared to placebo (Figure 3A). Overall LOXL2 activity was dose‐dependently reduced, with higher doses resulting in complete and more sustained inhibition (Figure 3B). Specifically, after a single dose of 100 mg, 80% inhibition of LOXL2 activity was evident at 4 h, with approximately 20% inhibition remaining at 24 h. This underscored the fast resynthesis of LOXL2 in humans, similar to that seen in rodents, with *ca*. 80% of enzyme activity returning within 24 h. At doses of 200 or 400 mg, PXS‐5338 achieved complete inhibition at 4 h, with 50% or 70% inhibition remaining at 24 h, respectively. These results tallied with the pharmacokinetic profile of PXS‐5338 at the different dose levels (Figure 3C), with drug concentrations approaching or above the (in vitro) IC_50,_ enabling target engagement (Figure 3D). Taken together, these results suggest that >200 mg of PXS‐5338 once‐a‐day would ensure continuous inhibition of LOXL2, despite its fast resynthesis, due to constant presence of the drug.

**FIGURE 3:**
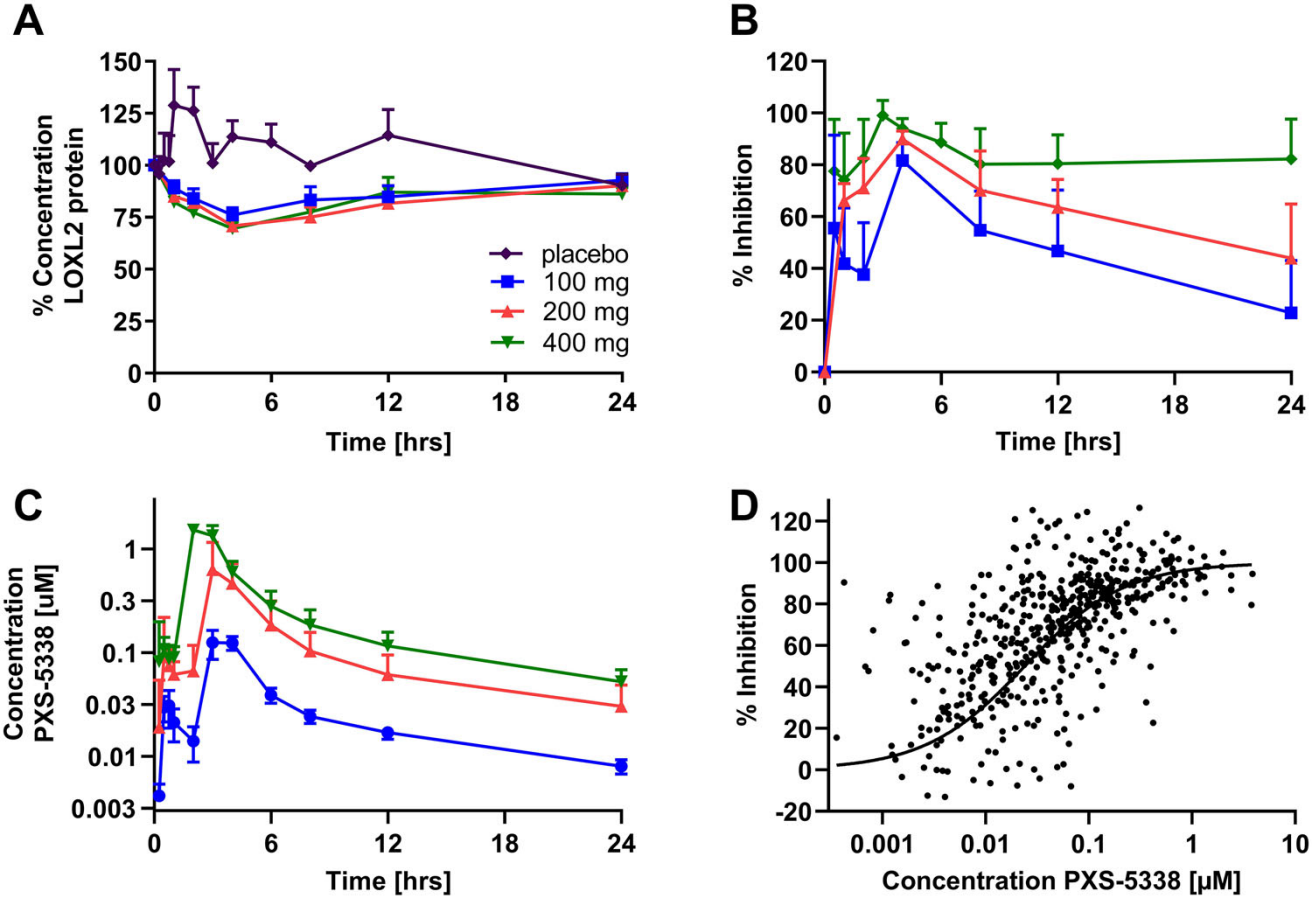
Inhibition of human lysyl oxidase‐like 2 (LOXL2) enzymatic activity by PXS‐5338 in clinical trials. (A) LOXL2 protein concentration in human serum is fairly constant over 24 h. Protein concentrations normalised to pre‐dose levels are plotted over time in healthy subjects treated with different doses (placebo, 100, 200 and 400 mg) of PXS‐5338. Mean ± SD with *n* = 6/time point. Phase 1 study with healthy male volunteers, ANZCTR ACTRN12617001444370. Legend of (A) also applies to (B) and (C). (B) Dose‐dependent inhibition of LOXL2 in plasma following a single oral dose of 100, 200 mg or 400 mg PXS‐5338. PXS‐5878/Simoa® platform used to measure the residual activity. Mean ± SD with *n* = 6/time point. (C) Pharmacokinetic (PK) profile of PXS‐5338 in healthy human males. The long‐lasting inhibition of LOXL2 activity by PXS‐5338 is driven by the prolonged compound half‐life in humans, with the irreversible nature of inhibition not significantly contributing to pharmacodynamics owing to the fast rate of de novo protein synthesis. (D) Correlation between LOXL2 protein concentration versus % inhibition. Activity data normalised to pre‐dose value. The calculated (correlation between LOXL2 protein concentration versus % inhibition) IC_50_ of 24 nM is in excellent agreement with IC_50_ values of 35 and 37 nM generated using recombinant and native human LOXL2, respectively; *n* = 210.

Our work underscores the importance of understanding both the target engagement properties of an inhibitor, as well as target resynthesis rate following inhibition, to enable an accurate and successful translation of preclinical data to clinical efficacy. In the case of simtuzumab it seems reasonable to suggest insufficient antibody concentrations, and low target inhibition, caused the clinical failures. The development of a bespoke ABP, PXS‐5878, enabled accurate measurement of the inhibition achieved by LOXL2 inhibitor PXS‐5338, as well as the rate of resynthesis of the enzyme. Moving forwards this knowledge will prove crucial for effective drug discovery and development efforts focussed on this important class of enzymes.

## CONFLICT OF INTEREST

All authors are current or former employees of Pharmaxis except Mary Brock and Christina Raso who are employees of Quanterix. All authors have read and approved the manuscript.

## Supporting information

Supporting information
