# Supplementary material for: An activity‐based bioprobe differentiates a novel small molecule inhibitor from a LOXL2 antibody and provides renewed promise for anti‐fibrotic therapeutic strategies
Source: Clin Transl Med. 2021 Nov 6;11(11):e572. doi: 10.1002/ctm2.572 (PMC8571951; doi:10.1002/ctm2.572)
Supplement: Supplementary file 1 — Supporting information [file CTM2-11-e572-s001.docx]

**Supplementary Information**





|  | **Recombinant human LOXL1 IC_50_, nM** | **Recombinant human LOXL2 IC_50_, nM** | **Recombinant human LOXL3 IC_50_, nM** |
| --- | --- | --- | --- |
| **PXS-5338** | **2530** | **35** | **385** |
| **Des-fluoro PXS-5338** | **17100** | **827** | **8110** |
| **PXS-5878** | **1770** | **203** | **973** |
| **Simplified des-fluoro analogue** | **nt** | **8000** | **nt** |
| **Reversible des-fluoro inhibitor** | **2470** | **464** | **698** |

**Supplementary Figure 1:** **Reversible** (**des-fluoro) inhibitors**

For fluoroallylamine-based inhibitors, replacement of fluorine with hydrogen (leading to the corresponding des-fluoro structures) results in a drop in potency and a switch from irreversible to reversible inhibition. (A) For PXS-5338, the corresponding des-fluoro analogue retains sufficient potency for LOXL2 to allow the (ir)reversible nature of inhibition to be accurately probed in a jump dilution assay. (B) For PXS-5878, removal of the fluoro moiety in a simplified analogue results in a significant drop in potency for LOXL2, meaning (ir)reversibility cannot be accurately measured. (C) Reversible inhibitor (lacking a fluoro group) with comparable potency to PXS-5878 and employed as a suitable positive control. nt: not tested.

For LOX and LOXL4 the activity of the protein is too low to conduct jump dilution experiments.

**Supplementary Figure 2: Validation of the LOXL2 capture antibody (AF2639)**

(A) Western blot evaluating the specificity of the LOXL2 capture antibody AF2639. Lane 1: molecular weights ladder, with adjacent labels. Lane 2: recombinant human (rh) LOX. Lane 3: rh LOXL1. Lane 4: rh LOXL2. Lane 5: rh LOXL3. Lane 6: rh LOXL4. Lane 7: native human LOXL2 from fibroblast cell culture; left: primary antibody (AF2639) and anti-goat secondary antibody (AB6741); right blot: secondary antibody control; note: here molecular weights ladder is Lane 7.

(B) Western blot analysis of IMR90 cells that underwent LOXL2 siRNA knockdown showing complete ablation of the LOXL2 band after transfection. Lane 1: molecular weights ladder. Lane 2: protein derived from IMR90 cells after mock transfection. Lane 3: protein derived from IMR90 cells after siRNA control (siControl) transfection. Lane 4: protein derived from IMR90 cells after LOXL2 siRNA transfection; left: primary antibody (AF2639) and anti-goat secondary antibody (AB6741); right blot: secondary antibody control.

(C) Representative images of immunofluorescence analysis of IMR90 cells with LOXL2 siRNA knockdown; left: control cells treated with siRNA control transfection agent show intense LOXL2 staining (red stain) in the extracellular domain; right: cells treated with LOXL2 siRNA show ablation of LOXL2; nuclear staining with Hoechst stain (blue).

**Supplementary Figure 3: Substrate (putrescine) competes with PXS-5338 for the active site of recombinant human LOXL2**

In the presence of increasing concentrations of the substrate (putrescine) the potency of PXS-5338 decreases, as both inhibitor and substrate compete for the lysine tyrosylquinone (LTQ) cofactor within the binding site. Assay is run in standard Amplex Red conditions but with different concentrations of the substrate added simultaneously with the inhibitor PXS-5338. Data represented as mean ± SEM with n = 3/concentration.

**Supplementary Figure 4: Collagen oxidation assay demonstrating poor efficacy of AB0023**

Comparison of rh LOXL2 activity using collagen as a substrate and with different concentrations of the LOXL2 antibody AB0023, its isotype control antibody GS834298, PXS-5338 and BAPN. The concentration response curves were almost identical between LOXL2 antibody AB0023 and its isotype control antibody. Data represented as mean ± SEM with n = 3/concentration. LOXL2 activity data devised from Amplex Red assay.

**
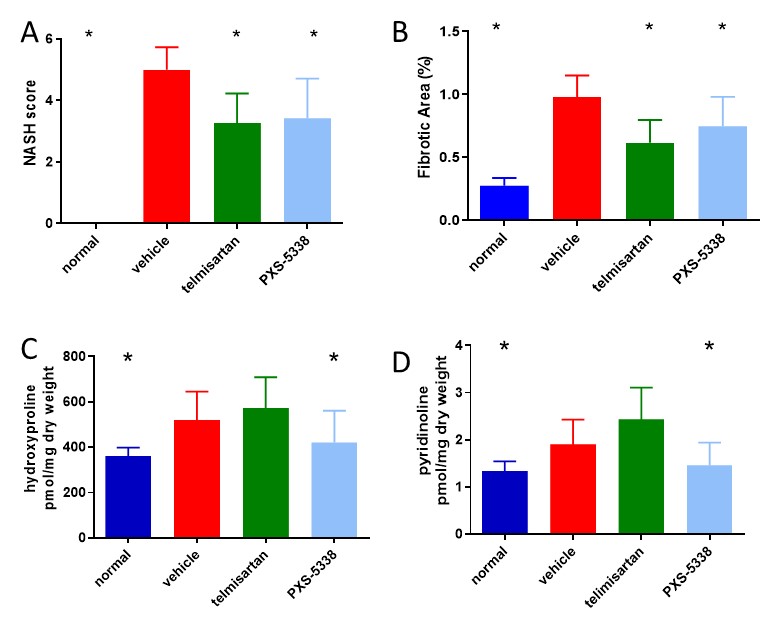
**

**Supplementary Figure 5: Anti-fibrotic effect of PXS-5338 in streptozotocin and high fat diet-induced model of NASH**

PXS-5338 was evaluated in a 14-week mouse model of NASH (Takakura, Koido et al. 2014), dosing therapeutically at 10 mg/kg from weeks 8-12 and increasing to 30 mg/kg from weeks 12-14. Histological analysis of the livers from the vehicle-treated NASH group exhibited micro- and macrovesicular fat deposition, hepatocellular ballooning and inflammatory cell infiltration compared with the sham control group. Consistent with these observations, NASH disease score significantly increased in the NASH group compared with the sham control group. PXS-5338 treatment significantly decreased NASH scoring compared with the NASH group. The improvement was also attributed to the reduction in hepatocyte ballooning, demonstrating the hepatoprotective effects of the compound. PXS-5338 significantly attenuated fibrosis, as observed by a reduction in fibrotic area (picrosirius red staining, B), as well as a significant reduction in collagen content (as measured by hydroxyproline, C) and a reduction in immature and mature crosslinks (as measured by DHLNL.not shown, and PYD, respectively, D). p<0.05 compared to NASH vehicle control with One Way ANNOVA, excluding telmisartan treatment group. Telmisartan served as a positive pharmacological control.

**Reagents**

PXS-5338 and PXS-5878 were prepared by chemical synthesis. The preparation of PXS-5338 is described in WO/2017/136871 and PXS-5878 was prepared using analogous methods. The purity of the compounds was >95% as determined by high performance liquid chromatography combined with mass spectrometry and proton nuclear magnetic resonance spectroscopy.

AB0023 and the corresponding isotype control antibody (GS834298 were kindly provided by Gilead). LOXL2 capture antibody AF2639 was from R&D Systems, the anti-goat secondary antibody secondary antibody (AB6741) and the second anti-LOXL2 antibody (AB213598) were from Abcam.

**Protocol for the detection of protein content and enzyme activity using Simoa^TM^**

The platform consists of an activity-based probe (ABP) for the detection of enzyme activity in conjunction with the Simoa^TM^ (single molecule array) bead technology to isolate the protein. This platform has enabled accurate measurement of total protein and enzymatic activity of LOXL2 from small volumes of serum, plasma and tissue.

In the immunoassay, paramagnetic capture beads are coated with a LOXL2 capture antibody (AF2639). 100 µL of pre-diluted (4-fold) sample in buffer (0.5% Casein, 0.25% Tween-20 in PBS) is combined in a cuvette with the capture beads (25 µL for protein content at 1.6 x 10^7^ beads/mL; 100 µL for enzyme activity at 4 x 10^6^ beads/mL). The biotinylated detector agent [either a second anti-LOXL2 antibody (AB213598) for protein content or the ABP (PXS-5878) for enzyme activity) is then added during the same incubation with intermittent shaking at room temperature (35 minutes for protein content or 54 minutes for enzyme activity).

Target molecules present in the sample are captured by the antibody coated beads and bound with the detector agent simultaneously. After incubation, beads are pelleted by magnetic separation, excess sample/buffer and reagents aspirated off and beads resuspended in wash buffer to remove unbound proteins and excess reagents.

Following protein capture and incubation with the detector, a conjugate of streptavidin-β-galactosidase (SβG; 100 µL at 300 - 350 pM) is mixed with the beads and incubated (5 minutes with intermittent shaking at room temperature). SβG binds to the biotinylated detector agent, resulting in enzyme-labelling of captured LOXL2. Following a final wash, the beads are resuspended in a resorufin-β-D-galactopyranoside (RGP) substrate solution and transferred to a Simoa^TM^ Disc (containing a microarray to separate beads into individual microwells). After settling into the microarray *via* gravity, beads are then sealed in microwells with oil. If LOXL2 is captured and labelled on the bead, β-galactosidase hydrolyses the RGP substrate in the microwell into a fluorescent product that provides the signal for measurement. A single-labelled target molecule results in sufficient fluorescent signal that can be detected and counted by the Simoa^TM^ optical system. The percentage of bead-containing wells in the array that have a positive signal is proportional to the amount of target or activity present in the sample.

To determine protein concentration, data can be interpolated from a calibration curve using rh LOXL2. For enzyme activity, samples are incubated for at least 30 minutes at room temperature with or without the pan-LOX inhibitor (100 μM BAPN). Data is presented as the ratio between the signal obtained in the presence (low signal control) and absence (high signal control) of BAPN. The activity unit is presented as a signal-to-noise (S/N) ratio and is considered to be the specific LOXL2 activity in the sample. All samples are measured in triplicate and the mean is presented per sample.
